# Highlighting the increasing need for anaesthetic support in interventional radiology

**DOI:** 10.1186/s42155-025-00628-w

**Published:** 2025-11-29

**Authors:** Jonathan Hanly, Hugo C. Temperley, Christopher O’Loughlin, Niall O’Sullivan, Marliza O’Dwyer, Richard Sweeney, Nazia Kahn, Robert Craig, Kevin P. Sheahan

**Affiliations:** 1 Department of Radiology, St James’s Hospital, Dublin, Ireland; 2 Department of Anaesthesiology, Children’s Health Ireland (CHI) at Crumlin Hospital, Dublin, Ireland; 3 Department of Anaesthesiology, Beaumont Hospital, Dublin, Ireland; 4 Department of Anaesthesiology, Guys and St Thomas’ NHS Foundation Trust, London, UK; 5 Hospital NHS Foundation Trust, University College London, London, UK

**Keywords:** Interventional radiology, Anaesthetic support, On-call services, Patient safety, Workforce challenges

## Abstract

**Introduction:**

The field of interventional radiology (IR) has witnessed rapid advancements, with an increasing emphasis on complex and high-risk procedures. Increasingly, new IR treatments are becoming both available and indicated for patients with complex comorbidities. As a result, anaesthetic expertise has become essential to ensure patient safety, optimise procedural outcomes, and manage perioperative complications. Despite this growing demand, dedicated anaesthetic teams in IR remain limited, which leads to concerns regarding patient safety and procedural efficiency.

**Methods:**

A comprehensive literature search was conducted across multiple databases, including PubMed, Embase, and the Cochrane Library, to identify studies published up to September 2024 that examined the expanding scope of IR, with a focus on the increasing need for anaesthetic support. This commentary draws on the existing literature to discuss the interaction between anaesthesia and interventional radiology, with particular attention to procedural sedation, pain management, and the care of high-risk patients. This commentary also evaluates workforce limitations, logistical challenges, and potential benefits of increased anaesthetic involvement in IR.

**Findings:**

Existing literature demonstrates the significant benefits of anaesthetic involvement in IR. The presence of an anaesthesiologist was associated with reduced procedural risks, enhanced patient satisfaction, and quicker postoperative recovery times. Despite these benefits, many IR departments remain under-resourced in terms of dedicated anaesthetic staff. Furthermore, training programmes for anaesthesiologists rarely focus on the unique demands of IR, creating a gap in specialist care.

**Summary:**

The growing complexity and risk associated with IR procedures underscore the need for expanded anaesthetic support in this field. Hospitals and healthcare systems should prioritise the integration of anaesthetic teams into IR, investing in specialist training and workforce expansion. Doing so can improve patient outcomes and the overall efficiency of IR procedures, reducing procedural risks, increasing patient satisfaction, and facilitating quicker postoperative recovery times.

## Introduction

Interventional radiology (IR) has undergone a significant transformation over the past three decades, with advances in imaging technology and endovascular techniques enabling the development of increasingly complex procedures [1, 2]. As IR interventions continue to become available and indicated for patients with complex comorbidities, the field has become a vital resource for delivering comprehensive patient care across various medical specialities.

The minimally invasive nature of these interventions can decrease the need for general anaesthesia, yet anaesthetic support remains crucial in maximising patient safety and procedural success [3]. The aim of integrating anaesthetic expertise in IR is the promotion of patient safety via pre-procedural patient assessment and optimisation, peri-procedural sedation and monitoring, and instituting post-procedural pain management protocols [4, 5]. Anaesthesiologists not only optimise personalised sedation and pain management but also can quickly respond to procedural complications as they arise [6].

This commentary explores the need for anaesthetic expertise in IR by examining its impact on patient care and workflow whilst identifying potential scheduling and training gaps. By outlining the essential role of anaesthesia in this rapidly expanding field, we highlight the need for integrated specialist practice to optimise patient outcomes.

Technically challenging procedures of longer duration are associated with an increased risk of fluid imbalance, electrolyte disturbance, hypothermia, hypoxia, and cardiovascular instability. As increasingly complex techniques develop, patients may feel heightened discomfort or anxiety, increasing the need for deep sedation or general anaesthesia [7].

The first edition of the ‘Provision of Interventional Radiology Services’, published by the Faculty of Radiologists and Radiation Oncologists, includes the standard of anaesthetic care required in the hospital setting. It recommends that each IR hub should have an anaesthetic nurse and anaesthesiologist available for out-of-theatre cases as required. Additionally, the hospital’s postanaesthesia care unit and staffing must be adequately equipped to care for patients afterwards [8]. These requirements set out the current position of IR within a modern healthcare system and aim to ensure sustainability and future services (Table 1).

**Table 1:** Roles of the anaesthesiologist in interventional radiology procedures

Pre-procedure	Intra-procedure	Post-procedure
Physiological optimisation for elective and emergency procedures	Safe provision of sedation	Multimodal analgesia guidance
Risk assessment	Conduct of general anaesthesia	Ongoing monitoring, resuscitation, and transport to a critical care environment
Medication planning	Haemodynamic monitoring	
Patient education	Resuscitation	

### Role of anaesthesiologists in interventional radiology

The field of IR is rapidly expanding, and there is significant variability in the involvement of anaesthesia during these procedures between centres [9, 10]. Given the rapidly evolving nature of IR, there is a lack of guidance and evidence defining the role of anaesthesiologists compared to more traditional operative environments [11]. However, the provision of anaesthesia in non-theatre environments is a long-standing core element of anaesthetic practice with extensive international guidance that can be readily applied to IR.

Evidence consistently demonstrates that the involvement of anaesthesiologists in IR procedures significantly reduces the risk of peri-procedural complications. Their contributions improve procedural efficiency and enhance the overall patient experience.

Schenker et al. (2009) found that general anaesthesia, managed by anaesthesiologists, led to fewer complications (including respiratory and cardiovascular issues) than sedation alone [12]. Apostolidou et al. (2022) emphasised that the presence of anaesthesiologists minimised complications, specifically in paediatric procedural sedation [13]. Tuite et al. (2005) emphasised the importance of anaesthesiologists in maintaining appropriate sedation depth and preventing complications, thereby contributing to safer procedures and smoother recoveries [14]. A study conducted by Wacker, J. et al. explored how anaesthesiologists contribute to faster recovery times by effectively managing sedation and monitoring during IR procedures [15]. The study also found that tailored anaesthesia techniques helped minimise side effects like nausea, vomiting, and dizziness, which can be detrimental to patient recovery.

Furthermore, a review of nonoperating room anaesthesia (NORA) in the USA emphasised how anaesthetic support contributes to more efficient IR procedures [16]. When anaesthesia teams are involved outside traditional operating room settings, procedures run more smoothly, with fewer interruptions.

### Conduct of anaesthesia

Anaesthetic support can range from the administration of anxiolytic medications in spontaneous breathing patients to a general anaesthetic with a definitive airway. There are significant risks and challenges associated with conducting anaesthesia in non-theatre environments [17].

The conduct of anaesthesia in any location must conform to the standards set out by international regulatory bodies. Minimum recommended monitoring involves pulse oximetry, a noninvasive blood pressure monitor, ECG, and monitoring of expired CO2/volatile anaesthetic agents, as well as airway pressure and temperature (if the intervention lasts longer than 30 min) [18]. For complex interventions or those with high-risk comorbidities, invasive arterial blood pressure monitoring and cardiac output monitoring may be indicated, allowing for an immediate response to haemodynamic fluctuations, whether due to an intervention complication or a deterioration in a patient’s chronic disease. The anaesthesiologist can manage haemodynamic fluctuations, arrhythmias, and resuscitation as required, allowing the interventional radiologist to appropriately focus on the primary issue (as opposed to attempting to focus on resuscitation and intervention simultaneously) [19].

The administration of procedural sedation necessitates close monitoring, as airway obstruction or apnoea can be disastrous if not recognised and promptly managed [20]. It is not usually deemed necessary for anaesthetic involvement with every case of procedural sedation. However, with the evolving nature of IR, a collaboration between specialities is imperative to assess patient's sedation requirements appropriately and plan accordingly. The anaesthesiologist may have an ongoing role in educating IR staff about the safe provision of procedural sedation and when anaesthesia involvement may be specifically warranted.

### Postoperative considerations

Anaesthesiologists have key expertise in the provision of acute pain management. When patients receive appropriate pain relief, they typically experience reduced anxiety, increased comfort, and faster recovery times — all of which lead to a more positive overall experience [3]. Research demonstrates that using multimodal analgesia effectively reduces opioid consumption and enhances patient satisfaction scores following IR procedures [21]. In addition, adequate post-procedural analgesia can lead to shorter hospital stays, thereby contributing to cost-effectiveness [22]. Multimodal analgesic regimens are tailored to the patient and the procedure. Specific IR procedures (such as those utilising ablation techniques) can have a significant analgesic burden. This was highlighted by Piccioni et al., who examined the significant pain associated with percutaneous ablation procedures [23]. Additionally, regional anaesthesia techniques, including neuraxial interventions (such as epidural or spinal anaesthesia), are also increasingly being utilised and can allow for reduced analgesic dosing, potentially further improving patient recovery [24].

## Current gaps and challenges

Evidence supports the role of anaesthetic involvement in optimising patient safety and outcomes in IR. However, the limited availability of anaesthetic support may pose a significant challenge. Although comprehensive data on this issue is limited, existing studies and observable trends offer important insights into the nature of this shortage and its potential impact on patient outcomes.

The Cardiovascular and Interventional Radiology Society of Europe (CIRSE) 2017 survey evaluated current European anaesthesia practices for IR [24]. Seventy-four per cent of respondents reported having access to a dedicated anesthesiologist. Notably, in 33% of cases, the reported availability of an anaesthesiologist was less than 10 h per week, with reported availability ranging from 8 to 24 h per week. Sedation was most frequently administered by an anaesthesiologist (48%), followed by an IR nurse (43%), and, in nearly 30% of cases, by an interventional radiologist. The reported variations in the availability of medical anaesthesiologists may be linked to the structure of the national healthcare system, funding strategies, and resource allocation, particularly in the context of demonstrable geographical trends [10].

A key challenge that must be addressed in the ongoing provision of anaesthesia services in IR is maintaining safety standards [17]. Basic safety standards, as stipulated by national bodies such as the ‘Difficult Airway Society’ (DAS) and the ‘Association of Anaesthetists of Great Britain and Ireland’ (AAGBI), must be adhered to. This includes minimal physiologic monitoring as outlined above, appropriately trained staff (including a dedicated anaesthetic nurse or assistant), appropriate supply and storage of equipment, and adherence to appropriate maintenance standards for this equipment [25]. A challenge often encountered in providing anaesthesia outside the operating theatre is unfamiliarity with the anaesthesia equipment utilised there [26]. To mitigate this, the IR suite must be appropriately adapted to conform to the same safety standards achieved in the operating theatre.

Other challenges faced in the IR suite are more complex to mitigate; these include smaller, more confined physical spaces and smaller teams that may be less familiar with the structured workflows associated with major operative interventions. Training programmes could focus on interdisciplinary courses integrating IR and anaesthesia. This may include simulation-based training, hands-on workshops, and clinical rotations tailored to interventional procedures. Additionally, developing certifications or fellowships that address the unique complexities and risks of anaesthetic management in IR could equip anaesthetists with the necessary skills and knowledge.

Operational challenges between the IR and anaesthesia departments frequently arise from scheduling conflicts, resource allocation constraints, and coordination inefficiencies, all of which can adversely affect the efficiency and safety of IR procedures [27]. Scheduling conflicts occur when both departments require access to shared resources, such as procedure rooms or clinical personnel, often resulting in procedural delays or cancellations [28]. Solutions include implementing flexible scheduling systems, dedicating specific time slots for IR procedures, and utilising centralised scheduling systems that better anticipate needs and prioritise urgent cases. Healthcare systems can conduct comprehensive needs assessments to understand the specific anaesthetic requirements for various IR procedures. They can then prioritise funding for staffing and resources, potentially reallocating budgets from less critical areas. Collaborating with hospital administration to emphasise the value of anaesthetic teams in patient outcomes may also help secure the necessary resources for expansion.

Resource allocation is another key challenge, as IR procedures require specialised equipment and anaesthesia resources. Studies demonstrate that the limited availability of both staff and equipment outside the operating theatre can cause delays [29]. Improved inventory management systems and shared resource pools between the IR and anaesthesia departments may enhance efficiency. Furthermore, aligning staffing models with the specific demands of IR, alongside cross-training personnel in both anaesthesia and IR procedures, can help ensure the consistent availability of essential resources [30].

As IR continues to evolve, the demand for dedicated anaesthetic support may rise, particularly as IR adopts an on-call service model. A shift toward 24/7 service delivery places additional pressure on both IR and anaesthetic teams, who must be equipped to provide prompt intervention to high-risk patients [31]. Without adequate anaesthetic support, patient safety during urgent, high-risk procedures may be compromised, potentially resulting in increased complication rates and poorer clinical outcomes [32].

## Summary

This commentary highlights the evolving nature of IR, which has expanded to include higher-risk procedures in elective and emergency settings. It describes the risks of providing novel interventional techniques to an ageing and multi-morbid population.

The commentary emphasises the critical role of anaesthesiologists in IR, highlighting how their expertise enhances patient safety and improves procedural outcomes. Their responsibilities range from optimising patients pre-procedurally to managing airway and haemodynamics during procedures, delivering general anaesthesia or procedural sedation safely, and overseeing recovery and ensuring effective postoperative pain management.

Whilst available evidence supports the benefit of anaesthetic involvement in IR, evidence for the consistent availability of this support remains limited. Further research is needed to support the funding and development of standardised integration models between anaesthesia and IR. Focus areas may include procedure-specific anaesthesia techniques, departmental staffing models, and the identification workflow barriers. Additionally, enhancing workforce capacity, multidisciplinary care models, and joint training may enhance collaboration between radiology and anaesthetic teams. This collaboration is crucial in maintaining patient safety and ensuring a sustainable future for the field of IR.

## Data Availability

Data sharing is not applicable to this article as no datasets were generated or analysed during the current study.
